# Lectures on Appendicitis and Notes on Other Subjects

**Published:** 1896-09

**Authors:** 


**Lectures on Appendicitis and Notes on other Subjects.** By Robert T. Morris, M.D. Pp. viii., 163. New York: G. P. Putnam's Sons. 1895.

The author thinks that a favourable time has come "for blazing one clear trail through the subject" of appendicitis, which occupies about half the volume, and he has really "blazed" very well. He knows what he wants to say and says it, tersely, clearly and forcibly. The book may be regarded as a concise account of the "American view" of appendicitis, and the author's style and opinions will be gathered if we quote from p. 36. "There is but one rule to be followed, and that is to isolate an infected appendix as promptly as we would isolate a case of diphtheria. . . . An infected appendix is isolated when it is out of the patient." On this side of the Atlantic most of us do not take quite so radical a view of appendicitis as those in America; but in spite of this we think—and we mean it as a compliment—that this is one of the most "pleasant" books we have read on this subject. The notes on various themes have not perhaps the same literary elegance; but they show that the author thinks and works on his own lines. The best of them are those which treat of Gall-stone Solvents, The Drainage Wick, and Subluxation of the Radius. There is a good index, some excellent illustrations, and the paper, printing and binding leave little to be desired.

